# Beyond social media users and game players: patterns of digital media use and their association with personality traits

**DOI:** 10.3389/fpsyg.2026.1643702

**Published:** 2026-02-13

**Authors:** Irena Stojković, Tatjana Mentus, Marija Jelić, Božidar Filipović, Marijana Veselinović

**Affiliations:** Faculty of Special Education and Rehabilitation, University of Belgrade, Belgrade, Serbia

**Keywords:** Big Five, digital media use activities, digital media use content, digital media use motivation, personality traits

## Abstract

**Introduction:**

Following the uses and gratifications theoretical framework, the objectives of the present study were to employ a person-centered approach to identify patterns of digital media use based on motivations, activities, and content, and to examine the role of personality traits in differentiating these profiles.

**Methods:**

The samples of high school and university students from Belgrade, Serbia, participated in Study 1, a qualitative focus group study aimed to investigate aspects of digital media use through qualitative content analysis; and Study 2, a quantitative study of constructing digital media use scales, establishing patterns of digital media use through latent profile analysis, and investigating their associations with personality traits.

**Results:**

The created scales of digital media use demonstrated adequate construct validity, reliability, and measurement invariance across gender. Seven distinct profiles of digital media use were identified. The profiles *High Social Media Users*, *Social Media Lurkers*, *Video Game Players*, and *Low Digital Media Users* align with previous research. Three novel profiles were established: *Science-Oriented Users*, *Creative Users*, and *Aggression-Oriented Users*. Discriminant analysis revealed that personality traits significantly predicted profile membership. A combination of high Openness and Extraversion primarily distinguished *Creative Users* from *Aggression-Oriented Users*, *Video Game Players*, and *Low Digital Media Users*. High Neuroticism combined with low Conscientiousness best differentiated *Aggression-Oriented Users* from *Science-Oriented Users*. Finally, a function primarily defined by low Agreeableness predicted membership in the *Aggression-Oriented Users* profile versus the *Social Media Lurkers* profile.

**Discussion:**

The findings offer implications for designing interventions that promote beneficial media use among young people.

## 1 Introduction

Digital media (e.g., the internet, social media, apps, and digital audio and video) are electronic technologies that use numerical codes to transmit information rapidly over long distances and provide users with greater opportunities for manipulation and creativity compared to older forms of media (Gaines, 2013). Drawing on various digital media theories, Delfanti and Arvidsson (2019) propose the following defining features of digital media: (a) convergent – various types of content, such as music, text, and video, can be produced and consumed on a single device, such as a smartphone or computer; (b) hypertextual – on the internet, texts that include references and links to other related content, enable users to easily explore information from multiple sources; (c) distributed – unlike traditional mass media, which are centralized and unidirectional, digital media allow all users to participate in the production and distribution of information; (d) pervasive – constantly present and widely accessible; (e) algorithmic – data on users’ behaviors and interactions on digital media platforms are collected and analyzed through algorithms and used to shape or influence future users’ experience; (f) asymmetric – corporations that own digital media services have the power to collect and analyze users’ data, while users themselves often lack knowledge about how algorithms function and shape their experiences; (g) ephemeral and permanent – although certain forms of content, such as messages or posts, may appear ephemeral to users, copies or traces of this content may be stored by other individuals or institutions for long periods. Since the 1990s, digital media have increasingly permeated daily life (Balbi and Magaudda, 2018), and today, they are used by most of the global population (Datareportal, 2025), through a wide range of digital devices, such as mobile phones, tablets, and laptops. While the gap in digital media use between younger and older generations is narrowing, usage remains especially high among youth, who have grown up immersed in these technologies (Eurostat, 2024). As a new form of media with numerous distinctive characteristics, digital media may carry specific implications for users’ psychosocial development. As a result, research on the relationship between digital media use and youth development is rapidly growing.

One aspect of digital media use that has been extensively studied is the motivation behind it. Uses and gratifications theory has formed the basis of most empirical studies in the field. According to the theory, personal needs and motivations shape expectations about media, which in turn lead to patterns of engagement that result in need gratification and other outcomes The theory assumes that: (1) persons have an active role in media use; (2) persons make media choices based on need gratifications expectations; (3) media compete with other forms of needs satisfaction; (4) persons are aware and may report their motives for media use (Katz et al., 1974). Building on this framework, Arnett (1995) proposed a typology of adolescents’ media use comprising Entertainment, Identity Formation, High Sensation, Coping, and Youth Culture/Subculture Identification. Entertainment refers to using of the media for enjoyment and for fun. Entertainment has been established as a motive for digital media use in numerous studies (e.g., Arness and Ollis, 2022; Gingras et al., 2025; Thorell et al., 2024). According to Arnett (1995), identity formation is the process of defining one’s values, abilities, and plans for the future, which occurs in part through engagement with various media content. Self-expression, which has been recognized as a motive for digital media use in previous research (e.g., Alsalem, 2019), is also a process that contributes to identity formation. Coping motivation refers to relieving and dispelling negative emotions. It is conceptually similar to escapism recognized in previous research on digital media use (e.g., Li et al., 2015; Merhi, 2016). High sensation seeking is a motive to experience novel and intense stimulation. Youth culture/subculture identification refers to a motive to engage with media in order to feel connected to a larger peer-group culture or subculture. The typology is developmental in nature, focusing on needs particularly relevant to youth, based on the developmental tasks of their life stage.

Subsequent empirical research on media use motivation has largely followed the uses and gratifications approach but has not drawn on Arnett’s typology. Numerous studies have investigated various motives for social media use. In a pioneering study, Joinson (2008) identified several uses and gratifications associated with Facebook use: social connection (reconnecting with and maintaining social ties), shared identities (joining groups, attending meetings, and connecting with like-minded individuals), content (posting and viewing photos), social investigation (engaging with application content), social network surfing (meeting new people and learning more about others), and status updating (updating one’s own status and following the newsfeeds of others). Subsequent research on motives for social media use has expanded this list, yet there is little consensus across studies regarding the number or types of motives identified. Nevertheless, motives commonly recognized as relevant for social media use include social connection, information seeking, escapism (from negative emotional states or boredom), entertainment, and self-presentation (e.g., Arness and Ollis, 2022; Gingras et al., 2025; Stockdale and Coyne, 2020; Thorell et al., 2024; Wei et al., 2024). Research has also examined the uses and gratifications associated with other forms of digital media. For online search, information seeking and Internet ambiance have been identified as primary gratifications (Thongmak, 2021). Regarding video games, studies highlight the relevance of enjoyment, fantasy and escapism, social interaction, self-presentation, and achievement motives (e.g., Li et al., 2015; Merhi, 2016).

Comparing Arnett’s typology, which serves as the theoretical framework of the present study, with the findings of previous empirical research, it can be observed that Entertainment and Coping, which are included in Arnett’s typology, are also identified in these studies. Furthermore, self-presentation, as discussed in prior research, may be conceptualized as a component of Identity Formation, as defined in Arnett’s typology. Social connection and information seeking, which are frequently identified in research on media use, are not included in Arnett’s typology. A possible explanation is that social communication and information seeking may be understood as activities that serve various underlying motives, such as entertainment, coping, or identity formation, rather than constituting distinct motivational categories. Furthermore, High Sensation and Youth Culture/Subculture Identification, which are included in the Arnett’s typology, have largely been neglected in previous empirical research on motivations for digital media use. Because Arnett’s typology is theoretically grounded in the conceptualization of the distinctive characteristics of the youth life period, it is particularly relevant as a framework for examining motivations for digital media use among young people.

Uses and gratifications approach posits that motivations shape activities and the choice of content persons engage with through media (e.g., Ruggiero, 2000; Rubin, 2002). By activities, we refer to the practices individuals perform while using digital media, such as messaging, posting, reading, or watching. By content, we refer to the subject matter of a text, message, post, or other material a person engages with (“Content”, Chandler and Munday, 2011). For example, if an individual posts a video of a birthday party, the act of posting represents the *activity*, whereas the birthday party depicted in the video represents the *content*. Digital media activities have garnered significant research attention. While earlier studies often employed aggregate measures of activities, it is now recognized that different digital media activities may lead to varying outcomes (Valkenburg, 2022). As a result, researchers have increasingly focused on examining the effects of specific activities—such as social media use, gaming, and video watching—on various aspects of psychosocial functioning (e.g., Gingras et al., 2023; Hygen et al., 2024; Kerr and Kingsbury, 2023; Svensson et al., 2022; Twenge and Farley, 2021).

The content individuals are exposed to or interact with through digital media is an understudied aspect of digital media use (Hancock et al., 2022). Research has largely focused on specific types of content linked to particular outcomes, such as social media portrayals of body ideals and their impact on body satisfaction (e.g., Castellanos Silva and Steins, 2023), violent video games and their relationship to aggression and prosocial behavior (e.g., Anderson and Bushman, 2001), or media depictions of risky behavior and risk-taking behavior (e.g., Khurana et al., 2019). However, a broader examination of the various types of content people engage with through digital media remains largely unexplored.

According to the uses and gratifications approach, media use is influenced by personal characteristics, among other factors (Rubin, 2002). A widely accepted model for understanding personality is the Big Five model, which conceptualizes personality through five traits: Neuroticism, Extraversion, Openness, Agreeableness, and Conscientiousness (McCrae and Costa, 2003). Neuroticism reflects the tendency to experience negative emotions. Extraversion relates to a preference for social interaction and activity. Openness indicates a receptiveness to new ideas and experiences. Agreeableness involves a selfless concern for others, including trust and generosity. Conscientiousness refers to traits like self-discipline, hard work, and achievement striving.

Personality traits may be conceived as basic tendencies that determine numerous aspects of person – environment interaction (John, 2021). They affect selection of environments, the way persons interpret the meaning of environments, as well as what aspects of these environments persons attend to. Furthermore, in interaction with environmental factors, personality traits exert an influence on various psychosocial outcomes (John, 2021). Thus, it may be assumed that they also affect individuals’ interactions with digital media, an assumption that has been empirically supported with regard to social media use and problematic media use(e.g., Ahmed and Ahmed, 2025; de Gil Zúñiga et al., 2017; Hawi and Samaha, 2019; Hidalgo-Fuentes and Fernández-Castilla, 2024; Peterka-Bonetta et al., 2021; Sánchez-Fernández and Borda-Mas, 2023; Wartberg et al., 2023). Regarding social media use, a literature review by Lampropoulos et al. (2022) shows that Openness and Extraversion are the strongest personality predictors of social media use, whereas Conscientiousness, Agreeableness and Neuroticism also predict this use, albeit to a lesser extent. A review study on the relationships between Neuroticism and social media use suggests that Neuroticism is associated with various social media activities and motivations. However, the findings across studies are often inconsistent. For example, individuals higher in Neuroticism tend to report excessive social media use in self-report measures, whereas objective measures of use do not support this association (Bowden-Green et al., 2021). Another review study on the relationship between Extraversion and social media use indicates that Extraversion is associated with an increased likelihood of social media use, time spent on these platforms, and the frequency of content creation (Bowden-Green et al., 2020). Research on the relationship between personality traits and other forms of digital media use beyond social media has been largely lacking in the literature. Further, research has revealed significant links between personality traits and various motivations for social media use, although the results of the studies are only partially consistent. For example, Chen and Peng (2023) found that the social interaction motives are positively related to Extraversion and Neuroticism; information-seeking and sharing motives are positively associated with Openness and Conscientiousness; expression and exhibition motives are positively predicted by Openness; escapism and relaxation motives are positively associated with Neuroticism; and norm- or trend-following motives are positively associated with Openness and negatively associated with Conscientiousness. Zhang et al. (2018) reported that social interaction and self-presentation motives are positively predicted by Agreeableness and Extraversion, and that self-presentation motives are negatively predicted by Conscientiousness.

A person-centered approach has been employed in several previous studies to identify distinct patterns of digital media use, that is, groups of young people characterized by specific configurations of media use variables. Patterns differing in the amount of the media use have been established (high, moderate or low rates of media use across various indicators). Besides that, patterns characterized by the high frequency of social media use and playing video games have emerged (e.g., Foerster and Röösli, 2017; Fredrick et al., 2025; Rideout, 2015; Song et al., 2023; Vannucci and McCauley Ohannessian, 2019). Patterns of high TV and video watching, and reading have also been reported (Rideout, 2015).

### 1.1 The present study

Previous research has typically examined the three key aspects of digital media use, motivations, activities, and content, in isolation. However, little is known about how these aspects interrelate to form distinct patterns of digital media use. This study advances prior work by adopting an integrative approach that simultaneously considers motivations, activities, and content preferences, thereby capturing multidimensional patterns of digital media engagement rather than treating these components as separate constructs. We argue that this approach provides a more comprehensive understanding of the diverse ways individuals interact with digital media. Further, by exploring the relationships between personality traits and various patterns of digital media use, this study aims to contribute to the literature on the role of personality traits in digital media use. Existing research has primarily focused on the role of personality traits in social media use, and problematic use of these media (e.g., Blackwell et al., 2017; Dilawar et al., 2022; Peterka-Bonetta et al., 2021; Zhang et al., 2018), neglecting their relevance to other forms of digital media engagement. In addition, this research advances the field by developing the first questionnaire assessing digital media use motivations grounded in Arnett’s (1995) typology of media use, alongside newly developed measures of digital media content and activities with established psychometric properties. This contribution is particularly important given the widespread use of measures whose psychometric characteristics have not been assessed. The present research is structured into two studies. Study 1 is a qualitative study based on focus-group interviews aimed at gaining insights into young people’s experiences with digital media use. The findings of Study1 informed construction of the digital media use questionnaires assessing motivations, activities, and content, in Study 2. Study 2 is a quantitative study examining patterns of digital media use and their associations with personality traits.

## 2 Study 1

Study 1 is a focus-group study designed to examine young people’s experiences with digital media, including their motivations for using these media, the activities they engage in, and the types of content with which they interact. As the affordances of digital media are rapidly evolving with ongoing technological developments, we consider it important to gain insight into digital media use from young people’s own perspectives in the contemporary moment, in order to incorporate these findings into the construction of questionnaire measures of digital media use in Study 2.

### 2.1 Method

#### 2.1.1 Sample

Focus groups comprised 45 participants: 15 secondary school students and 30 university students (24 females, 21 males). The secondary school students attended gymnasium (general, university-preparatory school), while the university students were studying in humanities or technical sciences.

#### 2.1.2 Procedure

The focus groups were conducted in spring 2024. Prior to conducting the focus groups, participants and the parents of underaged participants provided informed consent. They were informed about the purpose of the study, to gain insights into digital media use among youth, and were notified that the focus-group interviews would be audio-recorded, but that no identifying information would be used in any future presentation of the data. Three focus group interviews, each comprising 15 participants, were conducted by two of the study authors on school or university premises. The participants within focus groups knew one another, which was expected to facilitate open communication. Each interview lasted between 65 and 85 min, was audio-recorded, and subsequently transcribed. Students received a gift card worth approximately 4 euros for their participation. Study 1 and Study 2 were approved by the Ethics Board of the Faculty of Special Education and Rehabilitation, University of Belgrade.

#### 2.1.3 Instrument

A semi-structured interview format was employed, as less structured approaches are recommended for exploratory research (Morgan, 1997). In focus-group questions, we did not stress the difference between digital media (such as the internet) and digital devices (such as mobile phones), as we intended to use various terms we believed would be familiar to participants and would direct them to think about their digital media use. The introductory questions were: “How often do you use a mobile phone?”, “How often do you use a computer or laptop?”, and “How often do you use the internet?” These questions were included to direct participants’ attention to their digital media use. They were followed by key interview questions addressing activities, content participants engage with, and motivation for digital media use (Table 1). As Arnett’s (Arnett, 1995) typology of media use provided a theoretical framework for exploring digital media use motivations in the present study the questions referring to motivation were derived from that theoretical framework (questions 16–21, Table 1). Additional probing questions were used throughout the interviews to elicit further detail or clarification, in line with participants’ responses to the questions (Krueger and Casey, 2015). Toward the end of the interview, participants were also asked about perceived advantages of digital media compared with older media (e.g., print media and analog television), as well as potential risks associated with digital media use. These topics were not included in the present analyses, as the specific aim of this study was to gain insights into motivations, activities, and content, conceptualized as core aspects of digital media use in this research. Participants’ perceptions of the advantages and risks of digital media therefore fell outside the scope of the present analyses and may be examined in future work.

**Table 1:** Key focus-group questions by topic.

Topic	Questions
DMU activities	Think about yesterday and describe everything you did using digital devices and digital media, such as your mobile phone, the Internet, computer, and television. What do you usually use your mobile phone for? What do you usually use a computer for? What do you usually use the internet for?
DMU content	What do you browse on the internet? What videos do you watch? What texts do you read? What news do you read? What films or series do you watch? What websites do you follow? What video games do you play? Have you ever encountered aggression or cruelty on the internet? If so, how did that happen?
DMU motivation	What are the main motives for your use of digital media (internet, mobile phone, laptop, tablet, etc.)? What would you miss most if you could not use digital media? What else would you miss if you could not use digital media? It is considered that individuals sometimes use mobile phones, the internet, or social networks for entertainment. What are your thoughts on this? It is considered that individuals sometimes use mobile phones, the internet, or social networks to experience strong feelings, intense experiences. What are your thoughts on this? It is considered that individuals sometimes use mobile phones, the internet, or social networks to cope with stress and negative emotions such as sadness, anger, or fear. What are your thoughts on this? It is considered that individuals sometimes use mobile phones, the internet, or social networks to explore and define their future goals and values. What are your thoughts on this? It is considered that individuals sometimes use mobile phones, the internet, or social networks to express who they are. What are your thoughts on this? It is considered that individuals sometimes use mobile phones, the internet, or social networks to stay connected with youth cultures. What are your thoughts on this?

#### 2.1.4 Data analysis

Focus- group interviews were analysed using qualitative content analysis according to Schreier (2012). The analysis followed these steps: (a) selecting material relevant to gaining insights into digital media use activities, the content participants engaged with, and their motivations for digital media use; (b) constructing a coding frame; (c) dividing the material into units of coding; and (d) assigning these units to the categories of the coding frame. The first three steps - selecting the relevant material, developing the coding frame, and segmenting the material into units of coding - were conducted by one of the study authors. In constructing the coding frame, the main categories (activities, content, and motivations) were concept-driven, while the subcategories were developed in a data-driven manner based on what participants reported about these topics. The assignment of units of coding to the categories was performed by two study authors, who agreed on the assigned codes for more than 90% of the units of analysis. The disagreements were resolved through discussion.

### 2.2 Results

Digital media use activities reported by focus-group participants belonged to the following categories:

- Communicating (e.g., messaging, phone calls);
- Searching for information (e.g., browsing the internet, following forums);
- Watching video materials (e.g., videos, films, streaming series);
- Reading (e.g., reading books, texts, comics);
- Playing video games;
- Listening to music;
- Online shopping;
- Creating (e.g., writing texts, taking photos or making videos);
- Posting (e.g., uploading photos to social network profiles).

Participants in the focus groups reported the following types of content they engaged with through digital media:

- Educational content (e.g., mathematics problems, programming codes required for studies);
- Art (e.g., paintings, artistic videos);
- Personal development content (e.g., nutrition, interpersonal relations, exercise, fashion);
- Sport (e.g., athletics, basketball);
- Social themes (e.g., kidnapping);
- Politics (e.g., the Cold War);
- Philosophy;
- Religion (e.g., history of Christianity);
- Science (e.g., history, sociology, biomedicine);
- Aggressive content (e.g., malicious comments, rude messages).

The following categories of motivations for digital media use were expressed by focus-group participants:

- To communicate (e.g., to arrange going out together, to text with friends);
- To get informed (e.g., to access information easily and quickly);
- To entertain oneself (e.g., to fill free time);
- To cope with problems (e.g., to relax, to stop thinking about daily issues);
- To experience high sensations (e.g., to feel emotional excitement);
- To connect with youth culture (e.g., to learn what is popular among young people);
- To express identity (e.g., to express oneself through posts);
- To explore identity (e.g., to decide on future profession through online exploration; to define personal interests).

## 3 Study 2

The main objectives of Study 2 were to (a) identify profiles of digital media use based on individuals’ motivations, activities, and the content they engage with, and (b) examine how these profiles are associated with personality traits. Following the uses and gratifications theoretical framework (Katz et al., 1974), we assumed that digital media use is organized into distinct, theoretically meaningful patterns and that personality traits help predict these patterns.

### 3.1 Method

#### 3.1.1 Sample

Sample 1, used for exploratory factor analyses (EFAs) of the digital media activities, motivations, and content questionnaires, included 955 participants (74.3% female). Of these, 251 were secondary school students (vocational schools or a gymnasium) and 701 were university students (studying humanities or technical sciences). The mean age was 20.45 years (SD = 2.47), range15–29 years.

Sample 2, used for confirmatory factor analysis (CFA), latent profile analysis (LPA), and discriminant analysis (DA), consisted of 1,212 students (636 secondary school and 576 university students; 54% female). Secondary school students attended either vocational schools or gymnasium, while university students were enrolled in humanities, natural sciences, or technical sciences. The mean age was 17.98 years (SD = 2.64), range 14–29 years.

In terms of parental education, most fathers had completed secondary education (56.7% in Sample 1; 47.3% in Sample 2), while most mothers had completed university education (44.7% in Sample 1; 45% in Sample 2). A small minority of parents had completed elementary education (1.8% of fathers and 2.1% of mothers in Sample 1; 2.3% of fathers and 1.7% of mothers in Sample 2).

#### 3.1.2 Procedures

Data were collected from May to October 2024. Prior to data collection, written informed consent was obtained from all participants, as well as from a parent for each underage participant.

Participants completed questionnaires on their mobile phones or laptops during class time, either at school or on faculty premises, under the supervision of the study authors. As compensation, they received either a gift card valued at approximately 4 euros or course credit, depending on their institution.

#### 3.1.3 Measures

##### 3.1.3.1 Digital media use motivations (DMU-M) scale

The initial pool of 27 items for the questionnaire measuring digital media use motivations was developed based on Arnett’s (1995) typology of adolescent media use, as well as themes emerging from the focus-group interviews conducted in this study. Items were created to reflect Arnett’s proposed motivation types, using phrasing adapted from participants’ own expressions during the focus groups for some of the items. Exploratory and confirmatory factor analyses (see Results section) resulted in a final 22-item scale comprising five subscales aligned with Arnett’s (1995) typology: Entertainment (5 items; Cronbach’s *α* = 0.82); Identity formation (4 items; Cronbach’s *α* = 0.83); High sensation (4 items; Cronbach’s *α* = 0.84); Coping (4 items; Cronbach’s *α* = 0.88); Youth culture/subculture identification (5 items; Cronbach’s *α* = 0.87) Participants rated each item on a 5-point Likert scale (1 = not at all, 2 = mostly not, 3 = not sure, 4 = mostly yes, 5 = completely yes).

##### 3.1.3.2 Digital media use activities (DMU-A) scale

An initial pool of 23 items assessing digital media use activities was developed based on a review of existing instruments and data collected from focus-group discussions. EFAs and CFAs identified five subscales, comprising a total of 13 items: Playing video games (2 items, Cronbach’s *α* = 0.61); Communicating and web browsing (3 items, Cronbach’s α = 0.47); Browsing social media profiles (3 items, Cronbach’s α = 0.76); Editing one’s social media profiles (2 items, Cronbach’s α = 0.70.); Creating original content (3 items, Cronbach’s α = 0.62). Participants rated how frequently they engaged in each activity using digital media over the past six months, using a 5-point Likert scale (1 = never or almost never; 2 = about once or twice a month; 3 = about once or twice a week; 4 = every day or almost every day; 5 = several times a day).

##### 3.1.3.3 Digital media use content (DMU-C) scale

An initial pool of 52 items assessing digital media content was generated. Of these, 42 items were constructed to reflect content reported by focus-group participants, while 10 items were adapted from the EU Kids Online questionnaire (Zlamal et al., 2020) to capture engagement with aggressive content, including bullying behaviors. Using EFAs and CFAs (see Results section), the following subscales were identified, comprising a total of 37 items: Educational content (3 items, Cronbach’s *α* = 0.85); Art (5 items, e.g., Cronbach’s α = 0.80); Personal development (5 items, Cronbach’s α = 0.84); Physical appearance (4 items, Cronbach’s α = 0.91); Sport (4 items, Cronbach’s α = 0.91); Social and political themes (3 items, Cronbach’s α = 0.80); Religion and philosophy (3 items, Cronbach’s α = 0.64); Science (3 items, Cronbach’s α = 0.87); Agressive content (7 items, Cronbach’s α =. 89). Participants rated their content engagement over the past six months, using a 5-point Likert scale (1 = never or almost never; 2 = about once or twice a month; 3 = about once or twice a week; 4 = almost every day; 5 = every day).

##### 3.1.3.4 The BFI-20

The BFI-20 (Tucaković and Nedeljković, 2025) is a short, 20-item version of the Big Five Inventory (BFI-44; John et al., 1991). It includes four items for each of the five Big Five personality dimensions, assessed on a five-point Likert scale (1 = strongly disagree to 5 = strongly agree). In the present study, Cronbach’s α reliability coefficients were as follows: Extraversion, α = 0.75; Neuroticism, α = 0.70; Agreeableness, α = 0.64; Conscientiousness, α = 0.69; and Openness, α = 0.74.

#### 3.1.4 Data analyses

EFAs with direct oblimin rotation and CFAs were used in the construction of the scales of digital media use. As skewness and kurtosis measures suggested that some items deviated from normal distribution, EFA with principal axis method of factor extraction (Osborne, 2014), and CFA with robust maximum likelihood method of estimation were applied.

Multi-group CFAs (MGCFAs) were conducted to examine configural (equivalence in factor structure), metric (equivalence in factor loadings), and scalar (equivalence in item intercepts) invariances of the scales across gender.

Latent profile analysis (LPA) was applied to establish profiles of digital media use, and discriminant analysis (DA) to investigate their relations with personality traits. Prior to conducting DA, normal distribution of personality traits was confirmed based on skewness and kurtosis.

EFAs and DA were performed in IBM SPSS Statistics (Version 27). CFAs and LPA were performed using R Statistical Software (v4.4.3; R Core Team, 2025), with the *lavaan* package (Rosseel, 2012) for CFAs and the *tidyLPA* package (Rosenberg et al., 2021) for LPA.

## 4 Results

### 4.1 Exploratory and confirmatory factor analyses of the questionnaires

#### 4.1.1 Exploratory factor analyses

##### 4.1.1.1 DMU-M scale

The Kaiser-Meyer-Olkin measure of sampling adequacy (KMO = 0.93) and statistically significant Bartlett’s test of sphericity (χ^2^ = 15139.23; df = 351; *p* < 0.001) indicated the suitability of the initial item pool for factor analysis. The Kaiser-Guttman criterion (eigenvalue greater than 1) and Cattell’s scree test suggested that five factors accounting for 68.3% of the total variance should be retained. Upon inspection, it was found that five items had loadings below 0.50 on the corresponding factors. After eliminating these items, we reran the factor analysis. The Kaiser-Guttman criterion suggested four factors, while the Cattell’s scree test suggested five factors. As the latter was in accordance with Arnett’s typology, five factors solution explaining 71.4% of the total variance was retained. The content of items with high loadings on factors (see Table 2) suggest that the factors represent five motivations of digital media use defined in Arnett’s typology.

**Table 2:** EFA of the DMU-M scale: factor loadings of items (pattern matrix).

I use digital media…	Factor				
	1-HS	2-E	3-YCI	4-IF	5-C
… to connect with young people around the world.			0.665		
… to learn about what young people like.			0.716		
… to connect with people who share my interests.			0.872		
… to feel connected with young people who are like me.			0.884		
… to communicate with people who are like me.			0.725		
… to entertain myself.		0.702			
… to pass the time.		0.884			
… to feel good.		0.410			
… to avoid boredom.		0.772			
… to enjoy interesting content.		0.650			
… to express my values.				−0.863	
… to express my worldviews.				−0.897	
… to show others who I am.				−0.748	
… to set the goals I want to pursue.				−0.436	
… to evoke strong feelings.	0.766				
… to experience new things.	0.586				
… to experience strong excitement.	0.883				
… to experience strong sensations (like sounds and colors).	0.691				
… to relax.					−0.708
… to cheer up.					−0.829
… to get rid of stress and tension.					−0.944

HS, High sensation; E, Entertainment; YCI, Youth culture/subculture identification; IF, Identity formation; C, Coping; loadings smaller than 0.30 are not reported.

##### 4.1.1.2 DMU-A scale

The Kaiser-Meyer-Olkin measure of sampling adequacy (KMO = 0.80) and statistically significant Bartlett’s test of sphericity (χ^2^ = 3982.19; df = 253; *p* < 0.001) indicated that the initial item pool is suitable for factor analysis. The Kaiser-Guttman criterion suggested 6, and Cattell’s scree test suggested 5 factors. In accordance with the latter, EFA with the number of factors fixed to 5 was applied. After eliminating items that had loadings below.50 or cross-loadings, 13 items representing the following dimensions of DMU activities were retained: Playing video games, Browsing social media profiles, Editing one’s own social media profiles, Communicating and web browsing, Creating original content (results of the final EFA are presented in Table 3).

**Table 3:** Efa of the DMU-A scale: factor loadings of items (pattern matrix).

Item	Factor				
	1-BSM	2-COC	3-PVG	4-CWB	5-EMP
I play single-player video games.			0.747		
I play video games with or against others.			0.658		
I communicate with friends, acquaintances or family members.				0.633	
I browse the profiles of my friends and acquaintances on social media.	0.710				
I browse profiles related to specific topics on social media.	0.683				
I check out the profiles of celebrities on social media.	0.657				
I share and update information about myself on my friends-only social media profile.					−0.739
I share and update information about myself on my public social media profile.					−0.717
I browse the internet.				0.466	
I listen to music.				0.551	
I post content online that I’ve created myself, such as music, poetry, videos, or computer programs.		−0.594			
I write comments on articles from online newspapers, information portals, or forums.		−0.615			
I create textual content		−0.506			

BSM, Browsing social media profiles; COC, Creating original content; PVG, Playing video games; CWB, Communicating and web browsing; EMP, Editing one’s social media profiles; loadings smaller than 0.30 are not reported.

##### 4.1.1.3 DMU-C scale

The Kaiser-Meyer-Olkin measure of sampling adequacy (KMO = 0.90) and statistically significant Bartlett’s test of sphericity (χ^2^ = 28099.334; df = 1,326; *p* < 0.001) indicated the suitability of the initial item pool for factor analysis. The Kaiser-Guttman criterion (eigenvalue greater than 1) and Cattell’s scree test suggested that 11 factors accounting for 67.0% of the total variance should be retained. After eliminating items with low factor loadings, as well as three items related to entertaining content due to low reliability, an EFA was conducted on the remaining items. The analysis yielded 9 factors explaining 72.9% of the total variance (see Table 4).

**Table 4:** EFA of the DMU-C scale: factor loadings of items (pattern matrix).

Item	Factor								
	1-AG	2-PD	3-SP	4-A	5-E	6-SPT	7-PA	8-S	9-RP
I use apps or websites for studying.					0.698				
I look online for information I need for studying or homework.					0.961				
When I do not understand something in my studies, I search for additional explanations online.					0.751				
I visit virtual art exhibitions.				−0.516					
I watch videos related to art.				−0.686					
I create artistic content (e.g., poetry, paintings).				−0.682					
I post artistic content on my profile.				−0.682					
I read texts or books about art.				−0.690					
I search online for information to help improve my everyday life, such as tips on relationships, health, and nutrition.		0.474							
I listen to the podcasts on how to improve my everyday life, such as those about relationships, health, nutrition.		0.473							
I watch videos that teach me how to do things I need to do (like cooking, repairs, and decorating).		0.906							
I read advice related to what I need to do (like cooking, decorating, and repairs).		0.855							
I apply the knowledge I gain from the internet to everyday life.		0.516							
I look up information on the internet about physical appearance and beauty.							0.672		
I look up information on clothing online.							0.860		
I follow advice on how to look better.							0.855		
I look up information on fashion online.							0.872		
I watch videos of sports events.			0.906						
I follow sports news.			0.991						
I look up information about sports online.			0.973						
I post sports news on my profile.			0.474						
I read online newspapers or watch the news.						−0.606			
I follow political speeches or campaigns.						−0.792			
I watch videos about political events in our country or around the world.						−0.860			
I watch videos or read about religion.									−0.458
I discuss philosophy with other people online.									−0.401
I discuss religion with other people online.									−0.944
I read texts or books about science.								−0.697	
I watch videos about scientific topics.								−0.813	
I follow science-related pages on social media.								−0.762	
Someone treats me in a hurtful or nasty way via a mobile phone or the internet.	0.788								
I treat someone in a hurtful or nasty way via a mobile phone or the internet.	0.716								
Nasty or hurtful messages are sent to me via a mobile phone or the internet.	0.866								
Nasty or hurtful messages about me are passed around or posted where others could see.	0.914								
I am threatened via a mobile phone or on the internet.	0.900								
I am left out or excluded from a group or activity on the internet.	0.747								
I am forced to do something I do not want to do via a mobile phone or internet.	0.837								

AG, Aggressive content; PD, Personal development; SP, Sport; A, Art; E, Educational content; SPT, Social and political themes; PA, Physical appearance, S, Science, RP, Religion and philosophy; loadings smaller than 0.30 are not reported.

#### 4.1.2 Confirmatory factor analyses

CFAs were conducted to examine model fit of the digital media use scales. Model fit was evaluated using the criteria outlined by Hair et al. (2014), according to sample size and the number of items. For the DMU-M the DMU-A scales, the criteria are as follows: the Comparative fit index (CFI) or Tucker–Lewis index (TLI) above 0.92, the root mean square error of approximation (RMSEA) < 0.07 with CFI of 0.92 or higher, the standardized root-mean-square residual (SRMR) ≤ 0.08 (with CFI above 0.92). For the DMU-C scale, the criteria are as follows: CFI above 0.90, the root mean square RMSEA < 0.07 with CFI of 0.90 or higher, SRMR ≤0.08 (with CFI above 0.92). As shown in Table 5, CFA suggests adequate fit of the DMU-M, DMU-A, and DMU-C models to the data, in the whole sample as well as among females and males.

**Table 5:** Results of CFA for testing model fit and MGCFA for testing measurement invariance across gender of DMU-M, DMU-A, and DMU-C scales.

Scale	χ^2^ (df)	CFI	RMSEA	SRMR	ΔCFI	ΔRMSEA	ΔSRMR
DMU-M							
Overall model	1126.454 (199)***	0.935	0.061	0.055			
Across gender							
Females	716.969 (199) ***	0.936	0.061	0.059			
Males	656.641 (199) ***	0.931	0.063	0.056			
Configural	1373.610 (398) ***	0.934	0.062	0.055			
Metric	1389.624 (415) ***	0.934	0.062	0.056	0.000	0.000	0.001
Scalar	1489.135 (432) ***	0.928	0.062	0.058	−0.006	0.000	0.002
DMU-A							
Overall model	238.020 (55) ***	0.936	0.053	0.043			
Across gender							
Females	165.618 (55) ***	0.925	0.055	0.045			
Males	139.040 (55) ***	0.936	0.053	0.047			
Configural	304.658 (110) ***	0.930	0.054	0.043			
Metric	322.364 (118) ***	0.929	0.054	0.045	−0.001	0.000	0.002
Scalar	420.537 (126) ***	0.896	0.062	0.052	−0.033	0.000	0.002
DMU-C							
Overall model	2722.521 (593) ***	0.926	0.052	0.050			
Across gender							
Females	2077.485 (593) ***	0.909	0.058	0.058			
Males	1668.598 (199) ***	0.916	0.054	0.056			
Configural	3746.083 (1186) ***	0.912	0.056	0.056			
Metric	3888.704 (1214) ***	0.910	0.056	0.059	−0.002	0.000	0.003
Scalar	4128.382 (1242) ***	0.901	0.058	0.061	−0.009	0.002	0.002

***p < 0.001.

MGCFAs were conducted to test the measurement invariance of the scales across gender, following the criteria recommended by Chen (2007) for the given sample size. Specifically, metric noninvariance is indicated when a change of ≥ − 0.010 in CFI is accompanied by a change of ≥ 0.015 in RMSEA or ≥ 0.030 in SRMR; scalar noninvariance is indicated by a change of ≥ − 0.010 in CFI, along with a change of ≥ 0.015 in RMSEA or ≥ 0.010 in SRMR. As shown in Table 5, the MGCFA results support configural, metric, and scalar invariance across gender for the DMU-M, DMU-A, and DMU-C scales. Regarding DMU-A, although the change in CFI for scalar invariance slightly exceeded the recommended threshold, the accompanying changes in RMSEA and SRMR were below the cut-off values. Therefore, the results support scalar invariance of the DMU-A across gender.

### 4.2 Latent profile analysis

**Figure 1:**
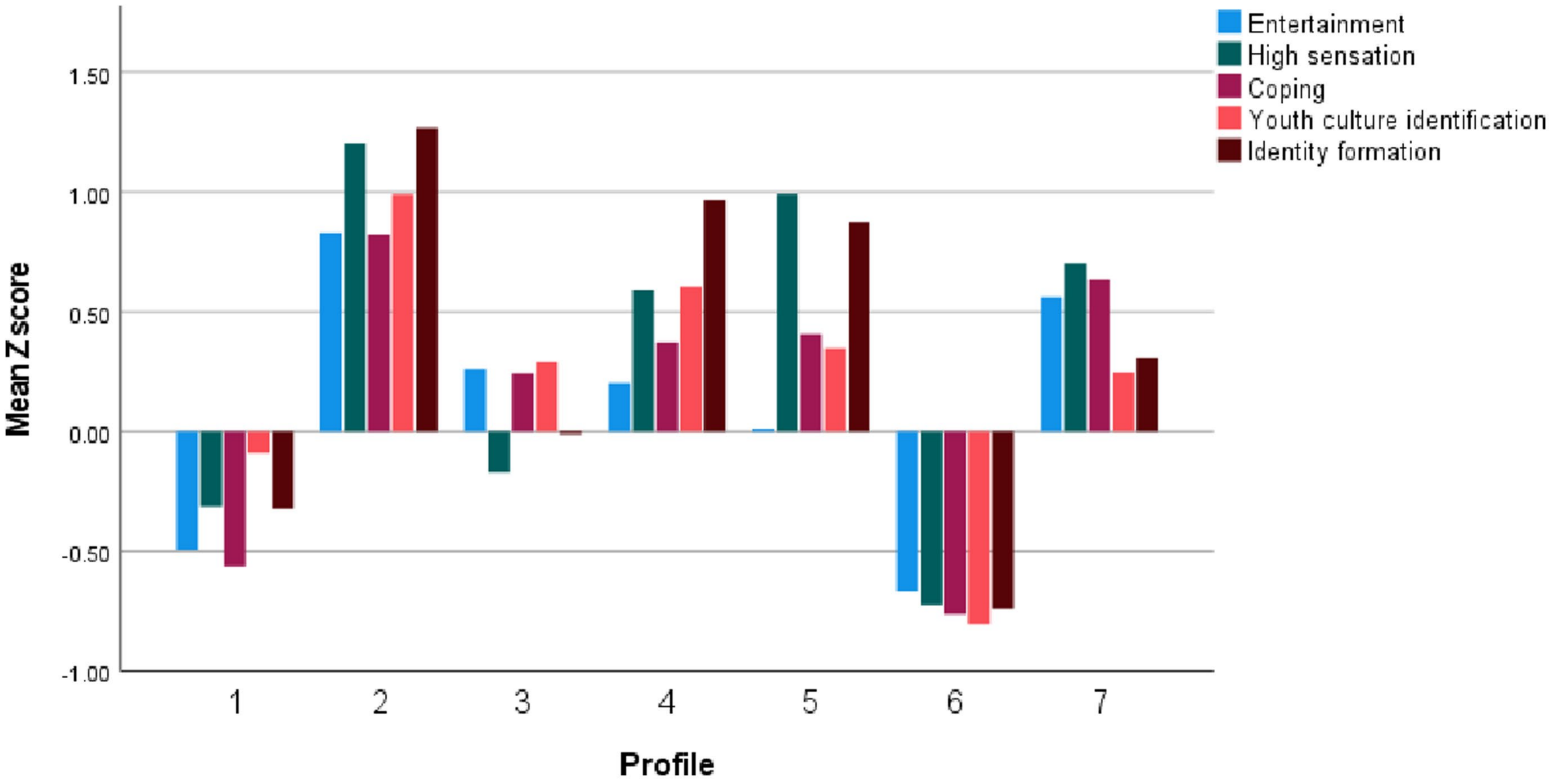
Digital media use motivations by profiles.

**Figure 2:**
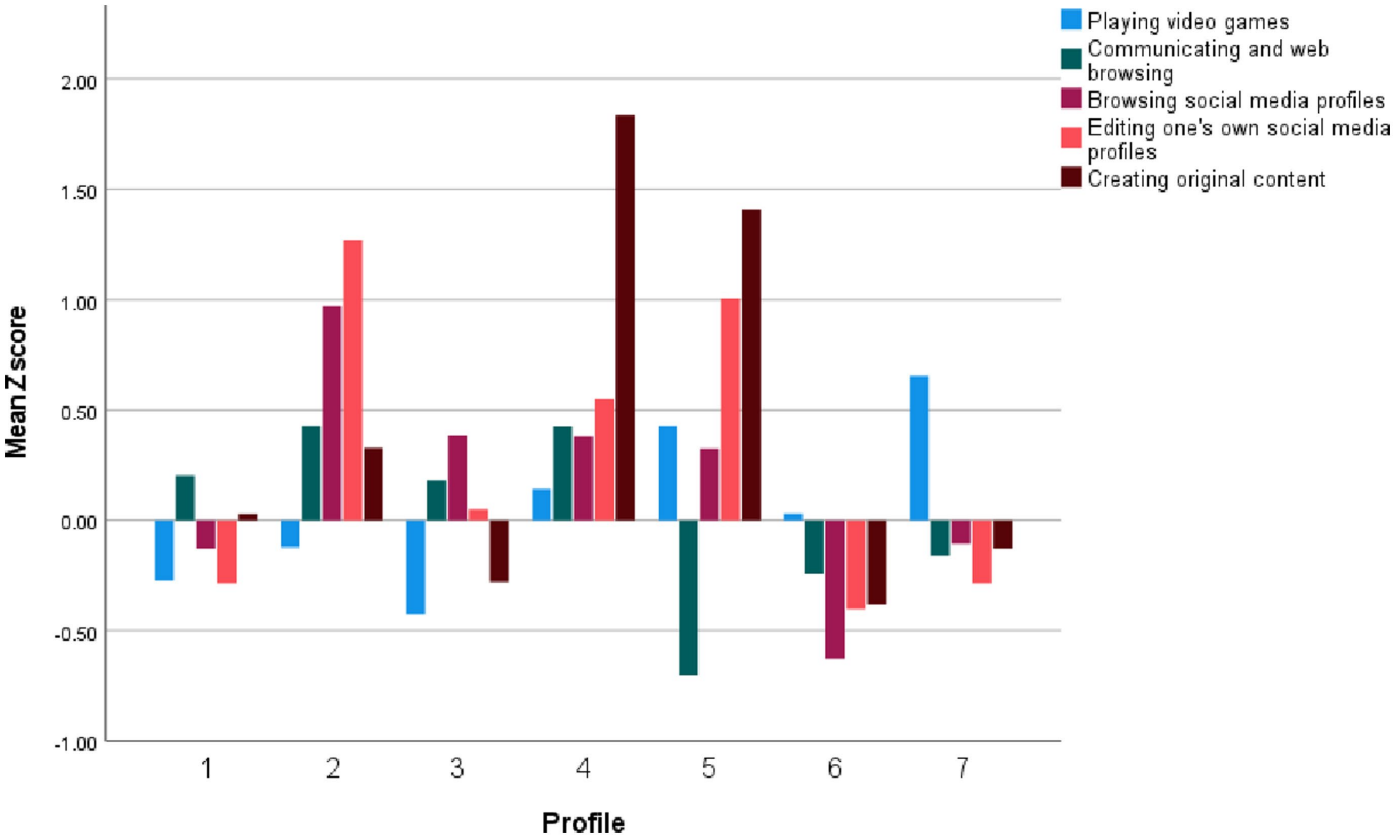
Digital media use activities by profiles.

**Figure 3:**
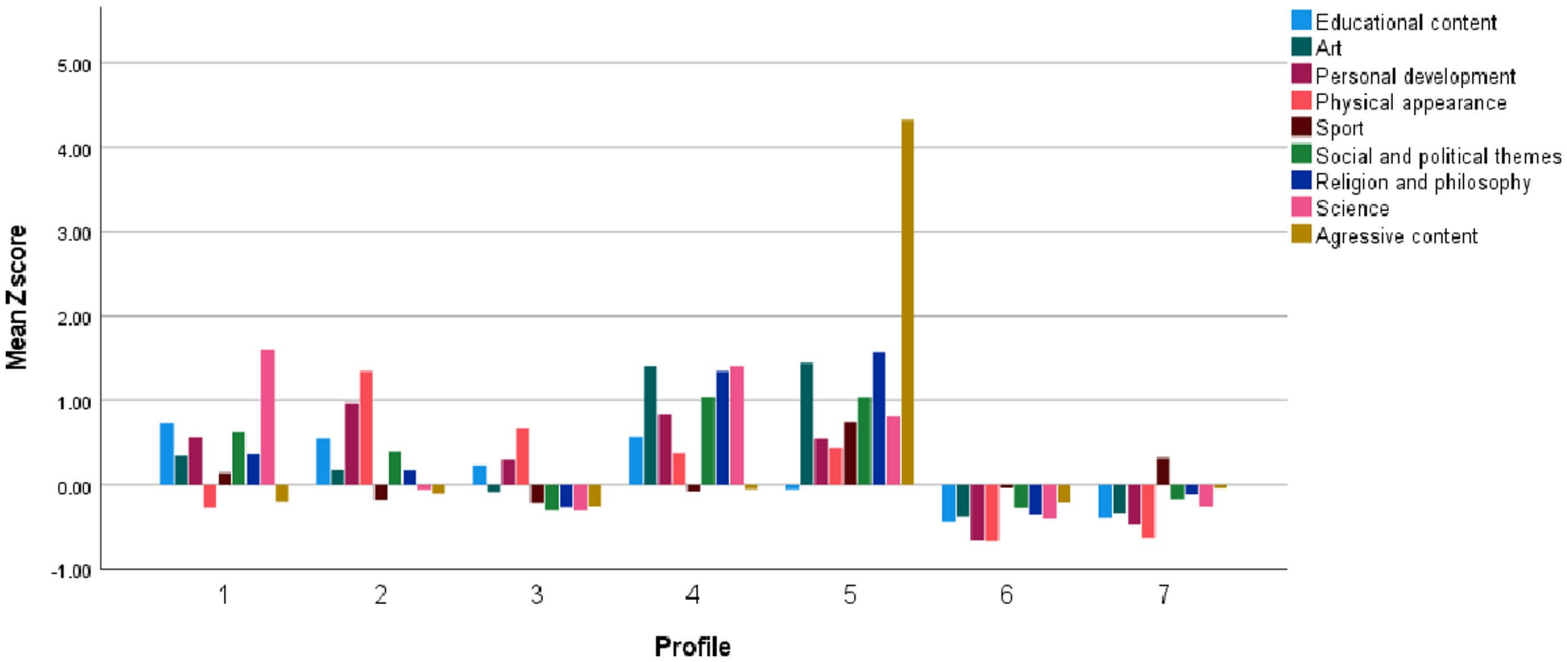
Digital media use content by profiles.

We conducted LPA based on scores from the DMU-M, DMU-A, and DMU-C subscales. We estimated Model 1 (variances set equal and covariances fixed at zero) and Model 3 (variances and covariances set equal) using one to eight latent profiles. To determine the optimal number of profiles, we considered the Akaike Information Criterion (AIC), Bayesian Information Criterion (BIC), and sample-size adjusted BIC (SaBIC). Lower AIC, BIC, and SaBIC values indicate better model fit. For Model 1, the best-fitting model according to AIC and SaBIC was the 8-class solution, whereas BIC favored the 7-class solution. For Model 3, the 8-class solution showed the best fit across all three indices. Upon reviewing the profile structures of the 7- and 8-class solutions, the 8-class solution was found to include two highly similar profiles characterized by low digital media use (i.e., low levels across all DMU subscales), with only slight differences regarding some DMU content subscales. Therefore, we selected the 7-class solution (Model 1) on the basis of greater parsimony and interpretability. Fit indices for Model 1 across one to eight classes are presented in Table 6. Standardized (z) scores on the DMU-M, DMU-A, and DMU-C scales by class are shown in Figures 1–3.

**Table 6:** Fit indices of the DMU latent profile solutions for model 1 with 1–8 classes.

Classes	AIC	BIC	SABIC	ENTROPY	BLRT_p
1	65407.6	65601.4	65480.7	1.000	0.01
2	63056.1	63351.9	63167.7	0.849	0.01
3	61823.4	62221.2	61973.4	0.843	0.01
4	60682.0	61181.8	60870.5	0.862	0.01
5	60383.6	60985.4	60610.6	0.820	0.01
6	60102.8	60806.6	60368.3	0.817	0.01
7	59853.5	60659.3	60157.5	0.825	0.01
8	59751.9	60659.7	60094.3	0.795	0.01

AIC, Akaike Information Criterion; BIC, Bayesian Information Criterion; SABIC, sample-size adjusted BIC; BLRT_p, *p*-Value for the bootstrapped likelihood ratio test.

The first profile (7.6% of the total sample; 7.0% of females; 8.5% of males) comprises individuals who exhibit moderate levels of Youth culture/subculture identification and moderately low levels on other motivational dimensions. They engage at moderate levels in Communication and web browsing, Browsing social media profiles, and Creating original content, and at moderately low levels in Playing video games and Editing one’s own social media profiles. Notably, these individuals report very high engagement with Science-related content, along with high engagement with Educational, Personal development, and Social and political content. We labeled this profile *Science-Oriented Users*.

The second profile (7.9% of the total sample; 11.5% of females; 3.7% of males) is characterized by very high levels of High sensation and Identity formation motivations, along with high levels across all other motivations. In terms of activities, individuals in this profile exhibit very high engagement in Editing one’s own social media profiles and Browsing social media profiles, a moderately high level of Communication and web browsing, and moderate engagement in other activities. These individuals show very high interest in Physical Appearance and Personal Development content, high interest in Educational content, and moderate interest in other content types. We labeled this profile *High Social Media Users*.

The third profile (26.6% of the total sample; 41.2% of females; 8.9% of males) is characterized as follows: in terms of motivation, individuals show moderately high levels of Youth culture/subculture identification, Coping, and Entertainment motivations, as well as moderate levels of High sensation and Identity formation motivations. In terms of activities, they engage at moderately high levels in Browsing social media profiles, at low levels in Playing video games, and at moderate levels in other activities. Regarding content, individuals in this profile report high interest in Physical appearance content and moderate interest in other content types. We labeled this profile *Social Media Lurkers*.

The fourth profile (7.0% of the total sample; 7.5% of females; 6.5% of males) is characterized as follows: in terms of motivation, individuals report a very high level of Identity formation, high levels of High sensation and Youth culture/subculture identification, and moderate levels of Entertainment and Coping motivations. In terms of activities, they exhibit very high engagement in Creating original content; moderately high engagement in Editing one’s own profiles, Browsing social media profiles, and Communicating and web browsing; and a moderate level of engagement in Playing video games. Regarding content, these individuals show very high interest in Art, Social and political themes, Religion and philosophy, and Science; high interest in Educational and Personal development content; and moderate interest in other content types. This profile was labeled *Creative Users*.

The fifth profile (3.9% of the total sample; 2.4% of females; 4.8% of males) is characterized as follows: in terms of motivation, individuals exhibit a very high level of High sensation, a high level of Identity formation, a moderately high level of Coping and Youth culture/subculture identification, and a moderate level of Entertainment motivation. Regarding activities, they show very high engagement in Creating original content and Editing one’s own social media profiles, moderately high engagement in Playing video games and Browsing social media profiles, and low engagement in Communicating and web browsing. In terms of content, these individuals report very high engagement with Aggressive content; slightly lower, but still very high interest in Art, Religion and philosophy, and Social and political themes; and high interest in other content types, except for Educational content, where they report moderate interest. This profile was labeled *Aggression-Oriented Users*.

The sixth profile (30.6% of the total sample; 24.6% of females; 38.0% of males) is characterized by low scores across all motivation scales. In terms of activities, individuals in this group report a moderate level of engagement in Playing video games and low levels of engagement in other activities. They exhibit low interest across all content types. This profile was labeled *Low Digital Media Users*.

The seventh profile (16.4% of the total sample; 5.8% of females; 29.5% of males) is characterized by high levels of Entertainment, High sensation, and Coping motivations, along with moderate levels of Youth culture/subculture identification and Identity formation motivations. Individuals in this profile report high engagement in Playing video games and moderate to moderately low engagement in other activities. In terms of content, they show a moderately high interest in Sports content, a moderately low interest in Physical appearance content, and moderate interest in other content types. This profile was labeled *Video Game Players*.

Key defining characteristics of the profiles are summarized in Table 7.

**Table 7:** Key defining characteristics (dominant motivations, activities and content) of the digital media use profiles.

Profile	Motivations	Activities	Content
Science-oriented users	Youth culture/subculture identification	Communication and web browsing Browsing social media profiles Creating original content	Science Educational content Personal development Social and political content
High social media users	High sensation Identity formation	Editing one’s own social media profile Browsing social media profiles Communication and web browsing	Physical appearance Personal development Educational content
Social media lurkers	Youth culture/subculture identification Coping Entertainment	Browsing social media profiles	Physical Appearance
Creative users	Identity formation High sensation Youth culture/subculture identification	Creating original content	Art Social and political content Religion and philosophy Science
Aggression-oriented users	High sensation Identity formation	Creating original content Editing one’s own social media profiles	Aggressive content Art Religion and philosophy Social and political content
Low digital media users	Low scores on all subscales	Low to moderate scores on all subscales	Low scores on all subscales
Video game players	Entertainment High sensation Coping	Playing video games	Sports

### 4.3 Discriminant analysis

DA was conducted to examine the contribution of personality traits to the differentiation of digital media use profiles. The independents-together method of variable entry was applied, and separate covariance matrices were used for the groups, as the significant result of Box’s M test indicated inequality of variance–covariance matrices (M = 146.602, *p* < 0.001; Brown and Wicker, 2000). Three statistically significant discriminant functions were identified (Table 8), with the first function accounting for a substantially larger proportion of the variance than the remaining functions. The first function is primarily defined by high levels of Openness and, to a lesser extent, high Extraversion. The second function is characterized by high Neuroticism combined with low Conscientiousness, while the third function is defined by high Agreeableness (see Structure Matrix, Table 7).

**Table 8:** Statistically significant discriminant functions generated by DA predicting DMU profile from personality traits, structure matrix and group centroids.

Variable/Statistic	Discriminant function		
	1	2	3
Wilk’s lambda	0.762***	0.892***	0.951***
% of variance	59.4	22.8	14.4
Structure matrix			
Openness	0.913	−0.273	−0.215
Neuroticism	0.153	0.757	0.164
Conscientiousness	0.136	−0.707	0.330
Agreeableness	0.155	−0.345	0.730
Extraversion	0.369	−0.056	0.295
Group centroids of profiles			
Science-oriented users	0.625	−0.530	−0.136
High social media users	0.384	0.218	−0.094
Social media lurkers	0.072	0.133	0.305
Creative users	1.014	0.131	−0.187
Aggression-oriented users	−0.321	0.596	−0.447
Low digital media users	−0.325	−0.231	−0.022
Video game players	−0.344	0.159	−0.160

***p < 0.001.

According to the group centroids (Table 7), the first function most clearly distinguishes *Creative Users*, who score at the positive pole, from *Aggression-Oriented Users, Low Digital Media Users*, and *Video Game Players*, who score at the negative pole. The function also positively predicts *Science-Oriented Users*, *High Social Media Users*, and *Social Media Lurkers*, with decreasing scores along that continuum. The second function primarily differentiates *Aggression-Oriented Users*, who score highly on this function, from *Science-Oriented Users*, who score at the negative pole. It also distinguishes other profiles to a lesser extent, with *High Social Media Users*, *Social Media Lurkers*, and *Video Game Players* scoring higher on this function compared to *Low Digital Media Users*. The third discriminant function differentiates *Social Media Lurkers*, who score at the positive pole, from *Aggression-Oriented Users*, who score at the negative pole.

## 5 Discussion

Following uses and gratifications approach, the objectives of the present study were to examine patterns of digital media use defined by motivations, activities, and content, and to examine their relationships with personality traits. Previous research has primarily employed a variable-centred approach, examining motivations for digital media use, activities, and content in isolation. To our knowledge, the present study is the first to investigate patterns of digital media use based on these three aspects concurrently. This approach provides a more comprehensive insight into the diverse ways digital media is used.

Following Arnett’s (1995) typology, we developed a scale to measure five types of digital media use motivations that he identifies as particularly important during the transition from childhood to adulthood. In addition, we created scales to assess digital media use activities and content. All scales demonstrated adequate construct validity and reliability, as well as measurement invariance across gender, making them a valuable tool for future research on digital media use.

### 5.1 Digital media use motivations, activities and content persons engage with

Regarding digital media use motivations, most focus-group participants identified communication and information access as their primary motives. In accordance with Arnett’s (1995) typology of media use, participants also described entertainment and coping as central motives. Other types of motivations in this typology - identity formation, high sensation seeking, and youth culture/subculture identification - were not mentioned spontaneously. However, when prompted, participants acknowledged that these motives were relevant to them and provided examples from their personal experiences. This suggests that although these motives may not be readily accessible or at the forefront of individuals’ awareness, they may nonetheless guide their digital media use. This assumption has received empirical support in previous research. For example, in relation to identity formation, Farrugia et al. (2019) report that adolescents use social networks for identity exploration. Sensation seeking has been found to be higher among Facebook users compared to non-users (Sheldon, 2012).

Communication and information seeking as motives for digital media use have also been recognized in previous research (e.g., Arness and Ollis, 2022; Thorell et al., 2024; Zhang et al., 2018). However, we elected not to include items representing these two motivations in developing the digital media use motivations questionnaire for two reasons. First, our aim was to construct scales derived specifically from Arnett’s (1995) typology. Second, as already noted in the Introduction, communication and information seeking may be understood as activities that potentially serve various motivations, rather than constituting motivational categories in their own right. Indeed, the dimension of communication and web-browsing activities included in the DMU -A questionnaire correspond to these motivations, rendering their inclusion in the motivations questionnaire redundant.

Regarding digital media use activities, focus-group participants reported a broad range of activities, indicating that young individuals interact with digital media through numerous practices. The types of activities identified in the focus-group interviews and incorporated into the DMU-A scale mostly align with those included in prior questionnaire-based measures of digital media use activities (e.g., Ma, 2018; Park and Lee, 2019).

With respect to digital media use content, the focus-group interviews also revealed a diverse array of content types that participants engage with. By incorporating these content categories into the DMU-C scale, this research offers an instrument that enables the assessment of the broad spectrum of digital media content individuals engage with, which has been largely absent from existing literature.

### 5.2 Digital media use profiles

We identified seven profiles of digital media use. One of these, the *Science-Oriented Users*, consists of individuals who show a particularly strong interest in scientific content, along with above-average interest in educational, personal development, and social and political content compared to other profiles. Their most prominent motivation for using digital media is youth culture identification. In terms of activities, they engage moderately in most types, except for editing their own social media profiles and playing video games, which are relatively low among this group. This profile comprises approximately 7% of the sample and includes a similar proportion of females and males.

As in previous studies that used activity-based approaches to establish user profiles (Foerster and Röösli, 2017; Fredrick et al., 2025; Rideout, 2015; Song et al., 2023; Vannucci and McCauley Ohannessian, 2019), a *High Social Media Users* profile was identified. In terms of activities, individuals in this profile display very high levels of editing their own social media profiles and browsing those of others—hence the name. They also exhibit moderately high levels of communication and web browsing, along with moderate engagement in other online activities. Our study extends existing knowledge of this profile by highlighting its unique characteristics in terms of user motivations and the types of content these individuals engage with. Compared to other profiles, *High Social Media Users* are characterized by very high levels of high sensation-seeking and identity-formation motivations, as well as elevated levels across all other motivation types. They also report a very high interest in content related to physical appearance and personal development, a high interest in educational content, and moderate interest in other content types. This profile is more common among females than males, with approximately 11% of females and 3% of males belonging to this group. Previous research has also found that a higher proportion of females belong to high social media use profiles compared to males (e.g., Foerster and Röösli, 2017; Fredrick et al., 2025).

The profile named *Social Media Lurkers* differs from the *High Social Media Users* profile primarily in that individuals in this group exhibit lower levels of editing their own social media profiles. They also score lower on other digital media use activities. Furthermore, their motivations for digital media use are lower compared to High Social Media Users. The largest differences are found in high sensation seeking and identity formation, which are both higher among High Social Media Users. Although *Social Media Lurkers* engage with various types of digital content in a similar pattern to *High Social Media Users*, they do so to a lesser extent. Consistent with our findings that these two profiles differ mainly in the extent to which users edit their own profiles, Winstone et al. (2022) also reported distinct social media use profiles that varied in content sharing and messaging, but not in profile browsing. In our study, the *Social Media Lurkers* profile includes the largest proportion of girls, approximately 41% of the sample, whereas it is much less common among boys, with about 9% belonging to this group.

The *Creative Users* profile is characterized by a very high level of creating original content. Individuals in this category also report moderately high levels of editing their own profiles, browsing social media profiles, communicating, and web browsing, along with a moderate level of video game play. They show high engagement with a broad range of content types, especially high engagement with art, social and political themes, religion and philosophy, and science. Additionally, they engage highly with educational and personal development content. These individuals are highly motivated to use digital media for identity formation. They also show strong motivations related to high sensation seeking and youth culture/subculture identification, along with moderate motivations for entertainment and coping. Approximately 7% of both females and males belong to this group.

The profile named *Aggression-Oriented Users* is characterized primarily by a very high level of engagement with aggressive content. Individuals in this profile also show very high relative engagement with artistic content, religion and philosophy, and social and political themes. They also score highly on all other types of content, except educational content, where their engagement is moderate. Motivationally, they are especially driven by high sensation seeking. They also report high motivation for identity formation, moderately high levels for coping and identification with youth culture, and moderate motivation for entertainment. In terms of activities, this group shows very high levels of creating and posting original content and editing their own social media profiles, moderately high engagement in video gaming and browsing social media profiles, and low levels of communication and web browsing. This profile comprises 2.4% of females and 4.8% of males in the sample.

The *Low Digital Media Users* profile is characterized by low scores across all motivation and content scales, along with generally low levels of digital media activity—except for a moderate level of video game play. Previous research using a person-centered approach to studying digital media use has also consistently identified a low-use profile (Foerster and Röösli, 2017; Fredrick et al., 2025; Rideout, 2015; Vannucci and McCauley Ohannessian, 2019; Winstone et al., 2022). Approximately 24% of females and 38% of males belong to this profile.

Consistent with previous research (Foerster and Röösli, 2017; Fredrick et al., 2025; Rideout, 2015;), a profile named *Video Game Players*, characterized by high levels of video game play, emerged in the present study. Individuals in this group display moderate to moderately low levels of engagement in other digital media use activities. Our findings suggest that they are primarily motivated by entertainment, coping, and high sensation seeking, and to a lesser extent by identity formation and youth culture/subculture identification. In terms of content engagement, they show above-average interest in sports content, below-average interest in physical appearance content, and about average engagement with other content types. Approximately 30% of males and 6% of females in the sample belong to this profile.

Previous research has demonstrated age-related differences across childhood, adolescence, and adulthood in forms of media use, such as social media and video game use (e.g., Hruska and Maresova, 2020; Politte-Corn et al., 2023; Ream et al., 2013). Following Arnett’s typology (Arnett, 1995), which emphasizes that the motivations it defines are particularly relevant for media use by young people, it can be assumed that patterns of digital media use change across development. Specifically, the levels of different motivational types, as well as the activities and content individuals engage with to satisfy these motivations, are expected to differ across age groups and to change over time within individuals. Accordingly, both between-person and within-person changes in digital media use patterns, as well as the factors influencing these changes, represent important avenues for future research. Furthermore, we propose that patterns of digital media use may prove to be stronger and more reliable predictors of various psychosocial outcomes than isolated measures of motivations, activities, or content persons engage with. This proposition should also be examined in future studies.

### 5.3 Personality traits as predictors of digital media use profiles

DA yielded three functions that predict digital media use profile membership. The first function reflects a combination of Openness and Extraversion, with a stronger association with Openness. *Creative Users* score highest on this function, followed by *Science-Oriented Users*, *High Social Media Users*, and *Social Media Lurkers*, who are positioned toward the positive end. *Aggression-Oriented Users*, *Low Digital Media Users*, and *Video Game Players* are located at the negative end of this function. This finding aligns with results from a cross-national study involving samples from 20 countries, which concluded that Openness and Extraversion are the strongest predictors of social media use (de Gil Zúñiga et al., 2017). A review study further supports this, noting a positive relationship between Extraversion and social media use (Bowden-Green et al., 2020). Our results offer a more nuanced view by showing that when social media use involves aggressive interaction—as observed among *Aggression-Oriented Users*—it is negatively associated with this same combination of traits.

The second function represents a combination of Neuroticism and low Conscientiousness. This function primarily differentiates *Aggression-Oriented Users* at the positive pole, from *Science-Oriented Users* at the negative pole. *High Social Media Users*, *Social Media Lurkers*, *Creative Users*, and *Video Game Players* are also positioned toward the positive end of this function, whereas the *Low Digital Media Users profile* is situated toward the negative end. In other words, high Neuroticism coupled with low Conscientiousness characterizes the *Aggression-Oriented Users*. Conversely, *Science-Oriented Users* tend to score highest on a combination of Conscientiousness and low Neuroticism. The finding that *Low Digital Media Users* are positioned toward the negative end of this function aligns with prior findings that low media use is linked to several positive psychosocial outcomes, such as higher well-being (Foerster and Röösli, 2017), increased school engagement, stronger classmate and teacher support, lower cybervictimization (Fredrick et al., 2025), greater family support, and fewer emotional and behavioral problems (Vannucci and McCauley Ohannessian, 2019). However, previous research suggests that low media use is not unequivocally beneficial as it may be related to less friend support and poorer friendship competences (Foerster and Röösli, 2017; Vannucci and McCauley Ohannessian, 2019).

The third function reflects Agreeableness and primarily discriminates *Social Media Lurkers* from *Aggression-Oriented Users*. Although the predictive power of this function is weak, it suggests that lower Agreeableness contributes to the likelihood of belonging to the *Aggression-Oriented Users profile*, along with lower levels of Openness, Extraversion and Conscientiousness and higher levels of Neuroticism.

### 5.4 Limitations and implications for future studies

Several limitations of the present study should be acknowledged. First, the DMU-A subscales Playing video games, Communicating and web browsing, and Creating original content, developed in the present study, exhibited low reliability. However, we retained them because they reflect important and widely prevalent activities among young people. The low reliability may indicate that these scales obscure the multidimensional nature of the underlying constructs. Consequently, the latent profiles identified may not reflect potentially distinct subgroups defined by different dimensions of these constructs. Future research could address this limitation by adding additional items to these scales to further explore their dimensionality and, if multidimensionality is confirmed, derive profiles that incorporate these dimensions. Moreover, while our study established metric invariance of the constructed digital media use scales across gender, their metric invariance across age should be examined in future research. Second, the sample consisted of individuals on academic tracks, which limits the generalizability of the findings. Digital media use profiles—and their associations with personality traits—may differ among working or unemployed youth. Future studies should examine potential differences in media use patterns between youth on academic tracks and those outside of them. Third, the cross-sectional design of this study precludes causal inferences between personality traits and digital media use profiles. The scarcity of longitudinal research on this topic in the existing literature represents an important gap that future work should address. Fourth, all constructs were assessed using self-report measures, which may have inflated the observed covariances. This limitation could be addressed in future studies by incorporating objective indicators of digital media use. Finally, an important limitation of this study is that the profiles of digital media use were established based on a sample of youth from Serbia. Given that culture shapes patterns of digital media use (Manago and McKenzie, 2022), the findings cannot be readily generalized to other cultural contexts. Therefore, future research should examine digital media use patterns with respect to motivations, activities, and content across diverse cultural settings.

## 6 Conclusion

By examining patterns of digital media use across activities, motivations, and content, we replicated previously identified profiles related to social media use, video gaming, and low media engagement. Our findings extend knowledge of these profiles by offering insight into motivations and content preferences of persons belonging to them. This approach also revealed three user profiles not previously documented in the literature: *Science-Oriented Users*, *Creative Users*, and *Aggression-Oriented Users*. An important direction for future research is to examine how these profiles relate to individuals’ psychosocial functioning. It may be assumed that the Science-Oriented and Creative User profiles are associated with positive functioning. If this is confirmed, interventions aimed at promoting these forms of digital media use could be developed. Conversely, the findings suggest that *Aggression-Oriented Users* should be a key target for interventions aimed at reducing the potential harms of this digital media use pattern, both for the users themselves and those they interact with.

## Data Availability

The datasets presented in this study can be found in online repositories. The names of the repository/repositories and accession number(s) can be found: Open Science Framework, https://osf.io/r2pg3/?view_only=807ebcebc7dc4b2c8d7914ec2551f954.
